# Conduction System Pacing With Defibrillator Therapy in a Patient With Heart Failure and Ventricular Arrhythmias After Mitral Valve Replacement

**DOI:** 10.7759/cureus.106736

**Published:** 2026-04-09

**Authors:** Mariola Szulik, Alexander Suchodolski, Ewa Jędrzejczyk-Patej, Wiktoria Kowalska, Radosław Lenarczyk, Michał Mazurek, Adam Sokal, Oskar Kowalski, Zbigniewa Kalarus

**Affiliations:** 1 Department of Cardiology and Electrotherapy, Silesian Center for Heart Disease, Zabrze, POL

**Keywords:** conduction system pacing, emergency echocardiography, holter monitoring, mitral valve replacement, ventricular arrhythmia

## Abstract

Heart failure with reduced ejection fraction (HFrEF) complicated by malignant ventricular arrhythmias remains a major therapeutic challenge, particularly in patients following complex mitral valve surgery. Conventional right ventricular pacing may aggravate ventricular dyssynchrony and further impair systolic function. Conduction system pacing (CSP) has emerged as a physiologic pacing strategy that directly activates the native His-Purkinje system, most commonly through His bundle pacing or left bundle branch area pacing (LBBAP), allowing near-normal ventricular activation. We report the case of a 70-year-old patient who developed severe heart failure, postoperative bradyarrhythmia, and an extremely high burden of ventricular arrhythmias following urgent mitral valve replacement. Despite pharmacological therapy and unsuccessful catheter ablation, electrical instability persisted. An implantable cardioverter-defibrillator (ICD) incorporating CSP using LBBAP was successfully implanted. Baseline QRS duration prior to pacing was 183 ms, whereas paced QRS duration after LBBAP implantation was 165 ms, consistent with near-physiologic ventricular activation. ECG documentation before and after device implantation demonstrated marked QRS narrowing and substantial suppression of ventricular ectopy after CSP-ICD implantation. Early post-implant monitoring demonstrated a marked reduction in ventricular ectopy and effective physiologic pacing. During 24 months of follow-up, pacing parameters remained stable, and no ventricular arrhythmias requiring ICD therapy were observed. This case demonstrates the feasibility of combining CSP with ICD therapy in selected patients early after mitral valve surgery. These findings are hypothesis-generating and require confirmation in prospective studies and larger clinical registries to define the broader clinical role and long-term durability of this strategy.

## Introduction

Heart failure with reduced ejection fraction (HFrEF) is associated with substantial morbidity and mortality and carries an increased risk of ventricular arrhythmias and sudden cardiac death. Ventricular ectopy and nonsustained ventricular tachycardia are frequently observed in patients with advanced heart failure and may become particularly pronounced in the setting of myocardial injury, postoperative stress, or progressive ventricular remodeling [1-3].

Conventional right ventricular pacing can induce electrical and mechanical dyssynchrony, potentially worsening left ventricular systolic function and contributing to pacing-induced cardiomyopathy. Cardiac resynchronization therapy (CRT) can mitigate dyssynchrony in appropriately selected patients, yet in unstable or early postoperative patients, the procedure may be limited by technical complexity, the need for coronary sinus lead placement, and concerns regarding lead stability and procedural burden.

Conduction system pacing (CSP) has emerged as a physiologic pacing strategy that directly activates the native His-Purkinje conduction network. CSP can be achieved using His bundle pacing or left bundle branch area pacing (LBBAP) [4,5]. By restoring physiologic ventricular depolarization, CSP may reduce dyssynchrony and preserve ventricular function. The 2023 European Heart Rhythm Association consensus statement recognizes CSP as an increasingly important pacing modality for both bradycardia and resynchronization indications [1].

Despite the growing body of literature on CSP, reports describing the combination of CSP with implantable cardioverter-defibrillator (ICD) therapy in patients early after valvular surgery remain limited [6,7]. This case report, therefore, describes the use of ICD therapy combined with LBBAP in a patient with severe heart failure, postoperative conduction disturbance, and refractory ventricular arrhythmias following mitral valve replacement.

## Case presentation

A 70-year-old male patient was admitted with rapidly progressive exertional dyspnea and markedly reduced exercise tolerance over the preceding two to three weeks, corresponding to New York Heart Association functional class III-IV. The patient had no previously documented history of coronary artery disease and no prior episodes of sustained ventricular arrhythmias.

Transthoracic echocardiography on admission revealed severe mitral regurgitation caused by posterior leaflet prolapse. The left ventricle was dilated with akinesia of the posterior wall and posterior papillary muscle region, and left ventricular systolic function was severely impaired, with a left ventricular ejection fraction (LVEF) of approximately 30%. Coronary angiography excluded significant epicardial coronary artery disease.

Because of rapid clinical deterioration and recurrent pulmonary edema, the patient underwent urgent mitral valve surgery. A 29-mm bioprosthetic valve was implanted in the mitral position, and the left atrial appendage was surgically closed.

**Figure 1:**
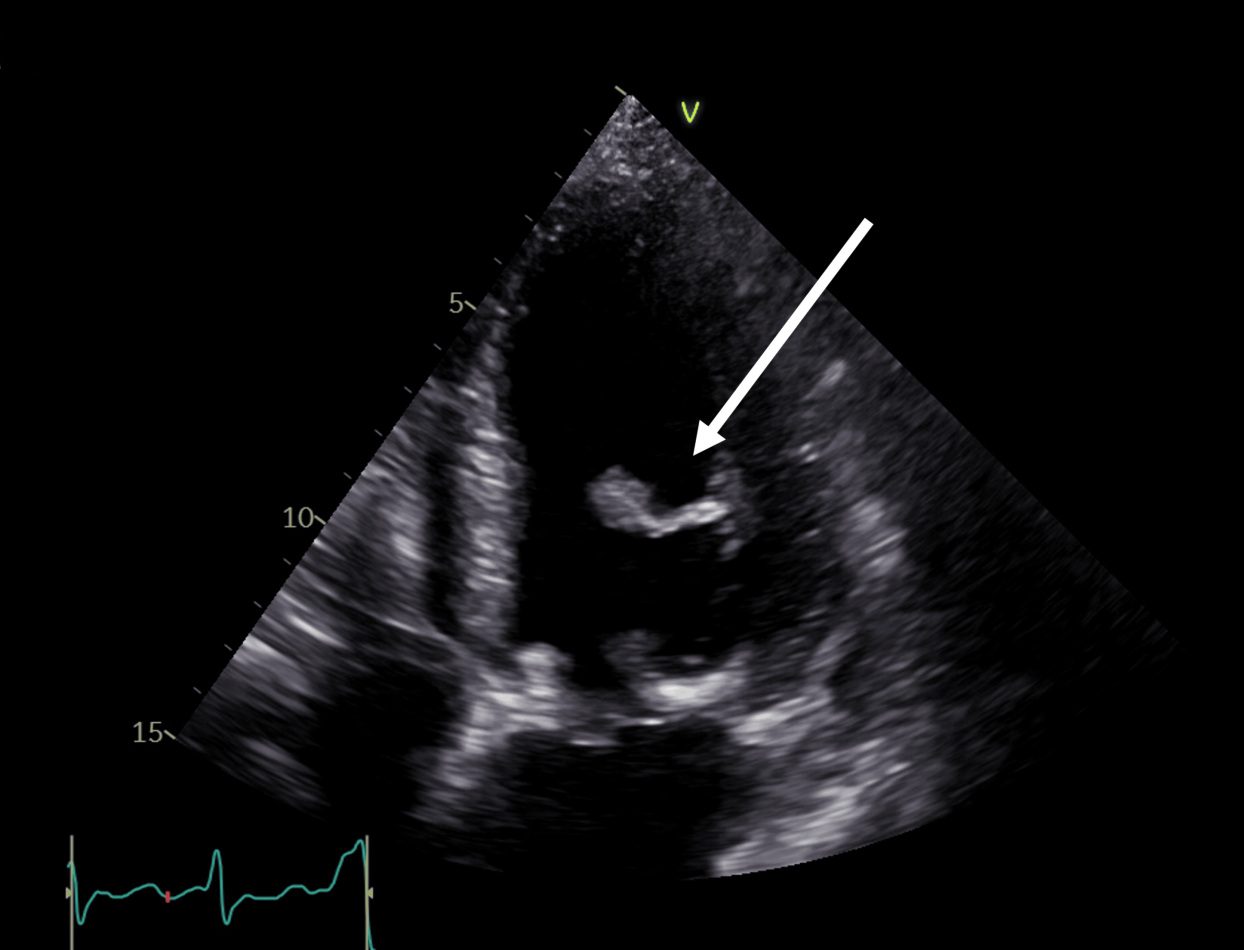
Transthoracic echocardiography showing papillary muscle rupture Apical four-chamber view reveals a ruptured papillary muscle within the left ventricle, contributing to severe postoperative mitral valve dysfunction and left ventricular systolic impairment

During separation from cardiopulmonary bypass, sudden cardiac arrest due to ventricular fibrillation occurred and required immediate direct defibrillation and resuscitation. A definitive single causative mechanism could not be identified. Ventricular arrhythmias occurring during separation from cardiopulmonary bypass are frequently multifactorial and may result from ischemia-reperfusion injury, electrolyte disturbances, catecholamine surge, or transient mechanical instability. Postoperative transthoracic echocardiography (Figure 1) demonstrated rupture of a papillary muscle within the left ventricle.

**Figure 2:**
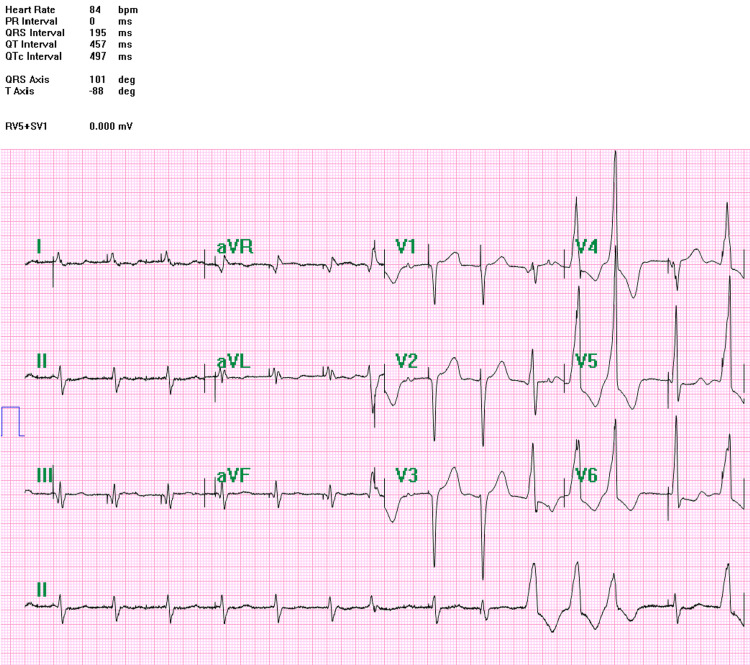
Early postoperative ECG showing frequent ventricular ectopic beats

Because of the extremely high operative risk, repeat surgical intervention was not pursued. The postoperative course was further complicated by severe bradycardia and recurrent ventricular arrhythmias requiring temporary pacing support. Twelve-lead electrocardiography obtained in the early postoperative period (Figure 2) revealed frequent ventricular ectopic beats consistent with a high arrhythmia burden. Holter ECG monitoring during the period of electrical instability documented 49,745 premature ventricular complexes, representing approximately 45% of all beats, together with 10,808 ventricular couplets and 106 episodes of nonsustained ventricular tachycardia.

Three-dimensional electroanatomical mapping was performed, and catheter ablation targeting ectopic activity originating from the right ventricular outflow tract was attempted. However, the procedure did not achieve sustained arrhythmia suppression. Serial echocardiography confirmed persistent severe left ventricular systolic dysfunction. LVEF was approximately 15% in the early postoperative period after mitral valve replacement and remained severely reduced at 14% at the one-year follow-up.

Pharmacological therapy was optimized during hospitalization and follow-up according to clinical tolerance and guideline recommendations. The patient was treated with guideline-directed medical therapy for heart failure, including metoprolol 200 mg daily, ramipril 2.5 mg daily, eplerenone 100 mg daily, and torasemide 20 mg daily. Sodium-glucose cotransporter-2 inhibitor therapy was not used because of a history of urinary tract infections. No major antiarrhythmic benefit sufficient to control the electrical instability was achieved with medical therapy alone.

Given the combination of advanced heart failure, postoperative bradyarrhythmia, severe LV dysfunction, and refractory ventricular arrhythmias, the patient was qualified for implantation of an ICD incorporating CSP via LBBAP.

Device Implantation

The CSP procedure was performed using a lumenless Medtronic SelectSecure™ pacing lead (Medtronic plc, Galway, Ireland) delivered through a dedicated sheath to the interventricular septum. The lead was positioned in the mid-septal region and advanced deep into the interventricular septum until capture of the left bundle branch area was achieved.

The implantation site was anatomically distant from the mitral prosthetic ring. Septal positioning was achieved without procedural difficulty, and no interference between the LBBAP lead and the recently implanted 29-mm bioprosthetic mitral valve was observed. At implantation, the pacing threshold was 0.5 V at 0.4 ms, R-wave sensing measured 10 mV, and lead impedance was 850 Ω.

**Figure 3:**
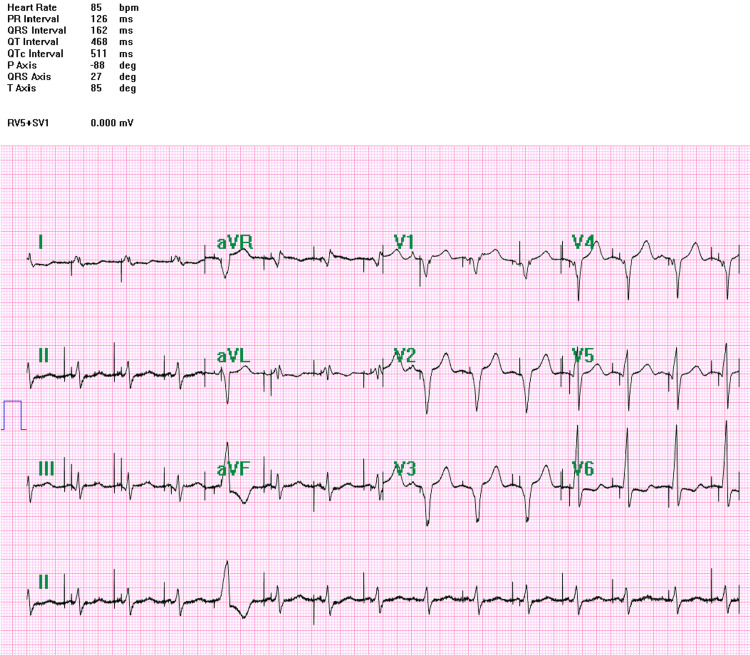
Post-implant ECG showing paced rhythm with narrow QRS complexes and absence of ventricular ectopic beats, indicating effective arrhythmia suppression

Left bundle branch capture was confirmed using established electrocardiographic criteria. The stimulus-to-left ventricular activation time (R-wave peak time in V6) measured 90 ms, and the V1-V6 interpeak interval was 28 ms, findings consistent with selective LBBAP. Baseline QRS duration prior to pacing measured 183 ms, whereas paced QRS duration following LBBAP implantation was 165 ms, indicating near-physiologic ventricular activation (Figure 3).

The ICD was programmed with standard ventricular tachycardia and ventricular fibrillation detection zones. Anti-tachycardia pacing was enabled in the ventricular tachycardia zone prior to shock therapy.

Follow-up

Early post-implant monitoring demonstrated 97% ventricular pacing and a dramatic reduction in ventricular ectopy to 251 premature ventricular complexes. During follow-up, pacing parameters remained stable, including threshold, sensing, and impedance values documented at 21 months after implantation. At the 24-month follow-up, the patient exhibited 94% CSP. Device interrogation revealed no sustained ventricular arrhythmias and no ICD therapies, including anti-tachycardia pacing or shock delivery.

Although a marked reduction in ventricular arrhythmia burden temporally followed CSP-ICD implantation, the clinical improvement should be interpreted cautiously, as postoperative recovery, rate regularization through pacing, and optimization of medical therapy may also have contributed to the observed stabilization.

## Discussion

This case illustrates the complexity of managing severe heart failure complicated by malignant ventricular arrhythmias in the early postoperative period following mitral valve surgery. Mechanical complications such as papillary muscle rupture and abrupt hemodynamic deterioration may create a highly arrhythmogenic substrate through acute ventricular volume overload, myocardial stress, and electrical remodeling.

Conventional right ventricular pacing may exacerbate ventricular dyssynchrony and further impair systolic function in patients with advanced heart failure, a finding consistently demonstrated in prior pacing studies and reflected in the development of pacing-induced cardiomyopathy. Although cardiac resynchronization therapy is beneficial in appropriately selected patients, it may be difficult to implement in unstable postoperative patients because of procedural complexity and the requirement for coronary sinus lead placement [4,8,9]. In contrast, CSP provides a physiologic pacing approach by recruiting the native His-Purkinje system and restoring a more natural ventricular activation sequence, which has been associated with improved electrical synchrony and favorable hemodynamic effects in observational studies [5,10,11].

Among CSP techniques, LBBAP has gained increasing attention because of favorable pacing thresholds, lead stability, and the ability to achieve rapid and synchronous left ventricular activation. Prior reports have shown that LBBAP may achieve a narrower paced QRS duration than RV pacing and may offer outcomes comparable to CRT in selected patients requiring resynchronization [5,10-12]. Our observation is consistent with these findings, as QRS duration narrowed from 183 ms to 165 ms after implantation, accompanied by stable long-term pacing parameters.

The electrocardiographic comparison between the early postoperative tracing (Figure 2) and the post-implant paced rhythm (Figure 3) visually supports the marked reduction in ventricular ectopy and improvement in ventricular activation. The marked reduction in ventricular ectopy after implantation is clinically notable. Similar reductions in ventricular arrhythmia burden after restoration of ventricular synchrony have been described in CRT populations, where improved electrical coordination may reduce dispersion of repolarization and arrhythmogenicity [8,10]. Whether a similar mechanism contributes to arrhythmia suppression during CSP remains uncertain but biologically plausible. In the present case, suppression was likely multifactorial and may also reflect postoperative stabilization, regularization of ventricular rhythm by pacing, optimization of heart failure therapy, and limited reverse remodeling.

This report also has educational value in highlighting a patient profile in whom CSP combined with ICD therapy may be considered: advanced HFrEF, severe postoperative ventricular arrhythmia burden, bradyarrhythmia or pacing requirement, and recent valvular surgery in which conventional CRT may be less attractive from a procedural standpoint. Because experience with CSP-ICD therapy in the immediate or early postoperative valvular setting remains limited, the present observation should be considered hypothesis-generating rather than definitive.

This report describes a single case and therefore has limited generalizability. Although follow-up extended to 24 months, longer observation is still required to evaluate the long-term durability of pacing parameters, ventricular function, valve-related interactions, and arrhythmia outcomes. In addition, potential confounding factors such as postoperative recovery and optimization of pharmacological therapy cannot be completely separated from the pacing intervention.

Furthermore, while no interference between the LBBAP lead and the mitral bioprosthesis was observed in this patient, careful assessment of individual anatomy and lead-to-valve relationships remains essential when considering this strategy after mitral valve surgery. Prospective studies and multicenter registries will be necessary to better define the role of CSP combined with ICD therapy in similar postoperative valvular populations.

## Conclusions

CSP combined with ICD therapy using LBBAP represents a feasible treatment option for selected patients with advanced heart failure, postoperative conduction disturbance, and refractory ventricular arrhythmias following mitral valve replacement. In this case, CSP-ICD implantation was associated with a substantial reduction in ventricular ectopy, stable pacing parameters during long-term follow-up, and absence of ventricular arrhythmias requiring ICD therapy at 24 months.

These observations are hypothesis-generating and should not be interpreted as proof of causality. Careful procedural assessment, including consideration of individual post-surgical anatomy and lead position relative to prosthetic structures, remains important when applying this strategy in similar patients. Further prospective studies are warranted.
